# A questionnaire of knowledge, attitude and practices on tuberculosis among medical interns in Nepal

**DOI:** 10.1016/j.jctube.2020.100173

**Published:** 2020-07-08

**Authors:** Anna Berg-Johnsen, Synne Osaland Hådem, Dipesh Tamrakar, Ingunn Harstad

**Affiliations:** a Department of Public Health and Nursing, Faculty of Medicine and Health Sciences, Norwegian University of Science and Technology, NO 7489 Trondheim, Norway; b Department of Pulmonary Medicine, St Olavs University Hospital, PO Box 3250 Sluppen, N-7006 Trondheim, Norway; c Department of Community Program, Dhulikhel Hospital, Kathmandu University Hospital, Dhulikhel, Kavre, Nepal

**Keywords:** Private medical college, Medical student, Tuberculosis control, Medical school, Stigma

## Abstract

**Background:**

Tuberculosis (TB) remains a major health problem worldwide, including in Nepal where around 33,000 new cases of TB were diagnosed in 2018 and 5400 patients died. There are challenges in the diagnostic process, treatment, and follow-up. Deaths, increased transmission and development of multi- drug resistant TB could be the consequences. Young doctors play an important role in this struggle, and therefore, their knowledge of and attitudes towards TB are crucial.

**Objective:**

We surveyed medical interns in Nepal regarding their knowledge, attitude and practices on TB and their adherence to the National Tuberculosis Programmes’ guidelines. The objective was to determine the associations between TB knowledge, and attitude and the factors that influence them.

**Methods:**

A WHO cross-sectional questionnaire template was modified and piloted. It was distributed anonymously among medical interns at three private medical colleges. Statistical analyses were performed to establish possible associations between TB knowledge and attitude, and the investigated variables, and to investigate differences between the medical colleges.

**Results:**

Of 270 interns, 185 (69%) interns were included. The mean knowledge score was 13,3 (SD: 2,12) of a maximum of 19. The possible attitude scores ranged from zero to 14 points, whereas the mean attitudes score was 9,4 (SD: 1,89). Some unacceptable attitudes and knowledge gaps were identified, including disease detection and management. There was an association between the knowledge score and attitude score and between the number of TB patients seen and knowledge/attitude.

**Conclusion:**

The surveyed interns had an adequate level of TB related knowledge, and acceptable attitudes. However, some unacceptable knowledge gaps and attitudes were detected. This survey underlines the considerable need of closing these knowledge gaps, and improving the attitudes, for which it is important for medical students to practice at a TB clinic and see a certain number of TB patients.

## 1 Introduction

Tuberculosis (TB) is still a major global health problem as the global incidence in 2018 was estimated to be 10.0 millions, and the mortality 1.2 millions [1]. The World Health Organization (WHO) and the United Nations’ Millennium Development Goals (MDG) [3] together with the Stop TB Strategy [4] have developed strategies for eliminating TB, which have led to a decline in absolute number of TB-deaths and TB incidence rate since year 2000.

Nepal is one of the member states that have committed to these strategies and have made progress regarding TB throughout the last decades. The National Tuberculosis Programme, (NTP) has been the responsible agency, and, in 1996, the directly-observed treatment short-course (DOTS) strategy, a 5-component strategy for TB management and control, was initiated in Nepal. Subsequently, the Stop TB Strategy, The WHO‘s strategy to curb TB by 2015, and the END TB Strategy were both adopted [5].

In 2018, 33.474.000 new cases were notified in Nepal, and an estimated 5.400 died from TB the same year [1], [6]. However, that figure likely underestimated the actual number of 8.000–10.000 cases, that were either not detected or not reported [6]. About 2.2% of new TB cases and 15.4% of retreatment cases have Multi Drug Resistant (MDR)-TB, with around 400 such cases reported each year. However, the real numbers could be higher as drug susceptibility testing is done only in a minority of TB cases. Health service delivery in Nepal is provided by both the private and public sectors. The NTP has faced challenges in providing free TB care and integrating the TB Control Programme into the private sector [7].

As TB is a leading cause of disability adjusted life years (DALYs), acknowledging, managing and investing in the disease will result in substantial economic and health returns [8]*.* WHO emphasizes that “the medical school should provide every graduate with the knowledge, skills and attitudes essential to the management of tuberculosis in the patient and in the community as a whole”[9]. Studies from several countries have assessed the knowledge, attitude and/or practices on TB among medical students and young doctors. Several of those studies indicate a lack of knowledge about TB among interns and inadequate management of the disease by them [10], [11], [12], [13], [14]. However, a study comparing students from Canada, India, and Uganda found that TB-related knowledge and practices were adequate [15]. The results were related to the number of TB patients seen and curriculum hours on TB. Another study from Italy also found a relationship between knowledge and the number of TB patients seen as well as a strong link between knowledge and memory of a previously taken Mantoux test [16].In this study, the knowledge was described as moderate. There have been no similar studies from Nepal.

The present study aimed to investigate interns knowledge, attitude and practices (KAP) on TB, as well as their adherence to the NTP guidelines. The purpose was to identify the associations between their knowledge and attitude and factors that influence them. This information can help make pre-and postgraduate medical teaching and training better suited to the needs of the population and the TB control programme. This is necessary to meet the future requirements for well-educated medical doctors with a good attitude towards TB patients.

## 2 Methods

This quantitative cross-sectional study was conducted via a self-administered, anonymous questionnaire. The questionnaire was a modified version of the WHO’s Knowledge Attitude Practice (KAP)-template assessing sociodemographic data and knowledge, attitude and practices on tuberculosis [17]. The questionnaire was piloted among 10 randomly selected young doctors in Nepal before the study could be conducted.

### 2.1 Study site and study population

Nepal has 19 medical colleges, and Kathmandu University (KU), a private university, has nine affiliated medical colleges. The Norwegian University of Science and Technology (NTNU) has a collaboration with the School of Medical Sciences (KUSMS) Dhulikhel at KU [18]. All KU-affiliated medical colleges were invited by a contact person at KUSMS to participate in the survey. Three colleges accepted the invitation: Nepal Medical College (NMC) in Kathmandu, Dhulikhel (KUSMS), and Bharatpur College of Medical Sciences (CMS) outside of the Kathmandu valley. The participants were medical interns who had completed their bachelor of medicine, bachelor of surgery (MBBS) degree**.** Participants were over 21 years old, and included both men and women.

Data collection took place in Nepal from 3 September 2017 to 17 October 2017. The interns who were available at their college at the time of the study; one day at CMS, and several days at KUSMS and NMC, answered a self-administered questionnaire on paper, which was handed out by a third party responsible for the interns at the respective colleges. The answers from the questionnaire were not scanned, but manually entered into SPSS Statistics Version 24 with every answer given a code during the entry. Data validity was ensured through double entry and crosschecking of data and random checks before analysis.

### 2.2 Analysis

SPSS Statistics Version 24 and Version 25 were used for the analyses.

Some of the questions allowed multiple responses (“check all that apply”), while other questions requiring only one answer. Some of the interns gave more than one answer to questions only requiring one answer. Those replies were excluded from the analysis.

The knowledge score was calculated based on 18 questions regarding TB knowledge. Correct answers were determined based on the annual report from the NTP in Nepal [6]. All questions with only one correct answer were each given 1point and questions allowing more than one answer were given 1–2 points. The maximum possible knowledge score was 19.

The attitude score to measure good or poor attitude towards TB was calculated based on attitude related questions. The maximum possible attitude score was 14 points. The answers indication a good attitude were given 2 points, while the answers showing poor attitude got zero points. Neutral and missing answers got 1 point. Question 32 regarding attitude allowed multiple responses. The choices were “fear”, “surprise”, “shame”, “sadness/hopelessness”, and “I don’t know”, with each choice getting 1 point. Thus, maximum score on this question was 4, while the other questions regarding attitude had a maximum score of 2 each.

To analyse the factors associated with the TB knowledge score and factors associated with the attitude score, regression analysis with one-way ANOVA was performed. To check correlation between knowledge and attitude two-tailed T-test using Pearson Correlation was used. Significance was considered at a p-value < 0.05.

### 2.3 Ethical considerations

The interns were given an information sheet and gave informed consent by participating in the study. The data was collected anonymously, and only unidentifiable sociodemographic data was collected.

A verbal request to the Regional Committees for Medical and Health Research Ethics Norway clarified that ethical approval in Norway was not needed as no patient information was gathered in the study.

In Nepal ethical approval was received from the KUSMS Institutional Review Committee (KUSMS/IRC) (84/17), and from the Research Director at NMC (IRC-NMC), while CMS accepted the KUSMS/IRC.

## 3 Results

### 3.1 Study population and characteristics

Three medical schools with a total of 270 interns accepted the invitation, with 185 (69%) interns, including 81 females (44%), participating in the study (Table1). The age of the participants ranged between 21 and 30 years, and 126 (68%) were below 25 years of age.

**Table 1:** Demographic characteristics of study population by medical colleges, gender and age.

		Medical colleges			Numbers
		KUSMS (%)	CMS (%)	NMC (%)	Total (%)
Total		70	90	110	270 (100)
Participated		63 (90)	44 (49)	78 (71)	185 (69)
Gender	Age				
Male		38 (60)	31 (70)	35 (44)	104 (56)
	<25 years	24 (63)	15 (48)	16 (46)	55 (53)
	≥25 years	14 (37)	16 (52)	19 (54)	49 (47)
Female		25 (40)	13 (30)	43 (55)	81 (44)
	<25 years	19 (76)	12 (92)	40 (93)	71 (88)
	≥25 years	6 (24)	1 (8)	3 (7)	10 (12)

KUSMS: Kathmandu University School of Medical Sciences, CMS: College of Medical Sciences, NMC: Nepal Medical College.

One out of five interns had taken a tuberculin skin test (TST) less than five years ago (Table 2), 131 (71%) had seen more than 10 TB patients, and 56 (30%) had friends or family members with TB (Table 2).

**Table 2:** Mean knowledge and attitude scores.

Variable		N (%)	Mean knowledge score (SD)	Mean attitude score (SD)
Overall		185 (100)	13.3 (2.1)	9.4 (1.9)
Gender	Female	81 (43.8)	13.9 (2.0)	9.6 (1.5)
	Male	104 (56.2)	12.8 (2.1)	9.3 (2.2)
Age	<25	126 (68.1)	13.5 (2.0)	9.2 (1.9)
	≥25	59 (31.9)	13.0 (2.3)	9.8 (1.9)
Medical college	KUSMS	63 (34.1)	12.9 (2.1)	8.6 (2.2)
	CMS	44 (23.8)	13.4 (2.0)	10.0 (1.4)
	NMC	78 (42.2)	13.6 (2.2)	9.7 (1.6)
Tuberculin skin test	≤5 yrs	37 (20.0)	14.4 (2.0)	10.0 (1.5)
	>5 yrs/never	148 (80.0)	13.1 (2.1)	9.2 (2.0)
No. TB patients seen	<10	54 (29.2)	12.5 (2.0)	8.7 (2.2)
	≥10	131 (70.8)	13.7 (2.1)	9.7 (1.7)
Friends or family with TB	Yes	56 (30.4)	13.8 (1.9)	9.5 (1.5)
	No	128 (69.6)	13.1 (2.2)	9.4 (2.0)

SD: standard deviation, KUSMS: Kathmandu University School of Medical Sciences, CMS: College of Medical Sciences, NMC: Nepal Medical College.

### 3.2 TB knowledge

**Fig. 1:**
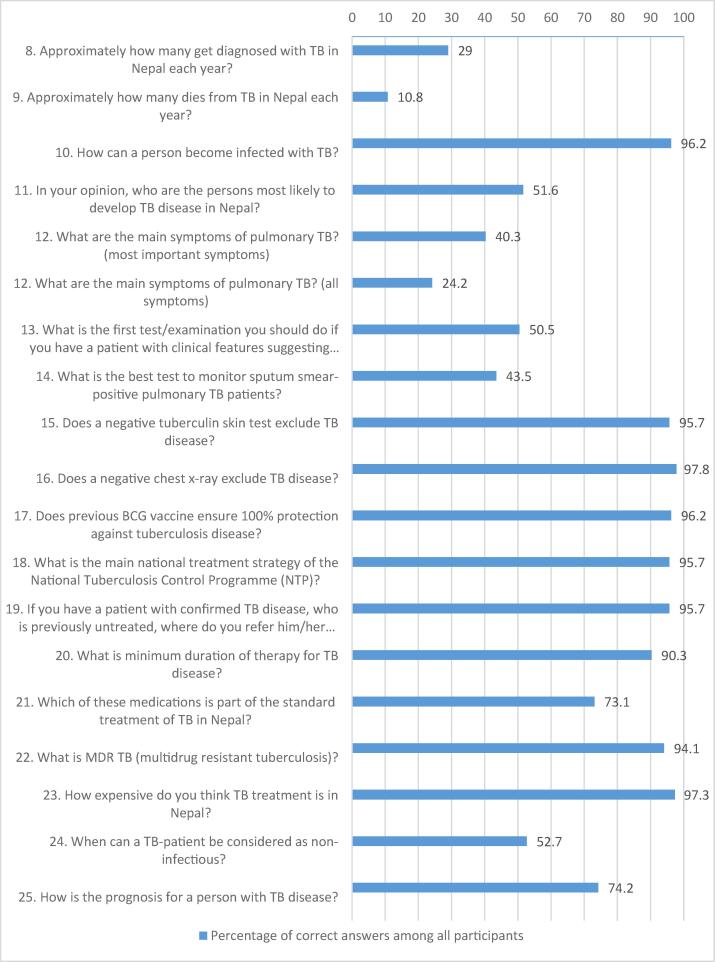
Percentage of correct answers on 18 knowledge-related questions regarding TB.

The survey found that 131 (71%) and 167 (89%) of the responders were unaware of the incidence of TB cases in Nepal and of TB related mortality (Fig. 1). Less than half of the interns mentioned guidelines or the NTP as the main source of information but almost all of them knew about the strategy for and costs of treatment. However, only one fourth of the interns knew all main symptoms of TB, and half of them knew the four most important symptoms. Also, half of them knew which test to do first and nearly half of them knew how to monitor TB. However, 178 (96%) knew about the referral process and knowledge about treatment was good. Only half of the interns knew that TB patients are non-infectious after two weeks of treatment (Fig. 1).

The mean knowledge score for all participants was 13.3 (SD: 2.12) of a maximum of 19. The mean knowledge score for the three medical colleges was as follows: KUSMS 12.9 (SD: 2.1) CMS 13.4 (SD: 2.0), and NMC 13.6 (SD: 2.2). There were no significant differences between the colleges. (Table 2, Table 3). However, there was a positive association between being female, having “seen more than 10 patients”, and having taken a TST within the last five years, and the knowledge score (Table 3). The interns who considered themselves not at risk for TB, or who would tell no one or just one person if they contracted TB, had a lower knowledge score.

**Table 3:** Correlates of TB knowledge, using one-way ANOVA.

	Variable	Estimate (95% CI)	P-value
Gender	Female vs. Male	0.9 (0.2 to 1.5)	0.010
Age	<25 vs. ≥25	0.1 (-0.7 to 0.6)	0.887
Medical college	Overall		0.584
	CMS vs. KUSMS	0.4 (-0.6 to 1.4)	1.000
	CMS vs. NMC	0.4 (-0.6 to 1.4)	1.000
	NMC vs. KUSMS	0.0 (-0.9 to 0.9)	1.000
Tuberculin skin test	≤5 yrs vs. >5 yrs/never	1.1 (0.3 to 1.9)	0.006
No. TB patients seen	≥10 vs. <10	0.8 (0.1 to 1.5)	0.022
Friends or family with TB	Yes vs. No	0.4 (-0.3 to 1.1)	0.235

TST: Tuberculin skin test, CI: Confidence interval, KUSMS: Kathmandu University School of Medical Sciences, CMS: College of Medical Sciences, NMC: Nepal Medical College.

### 3.3 TB attitude

**Fig. 2:**
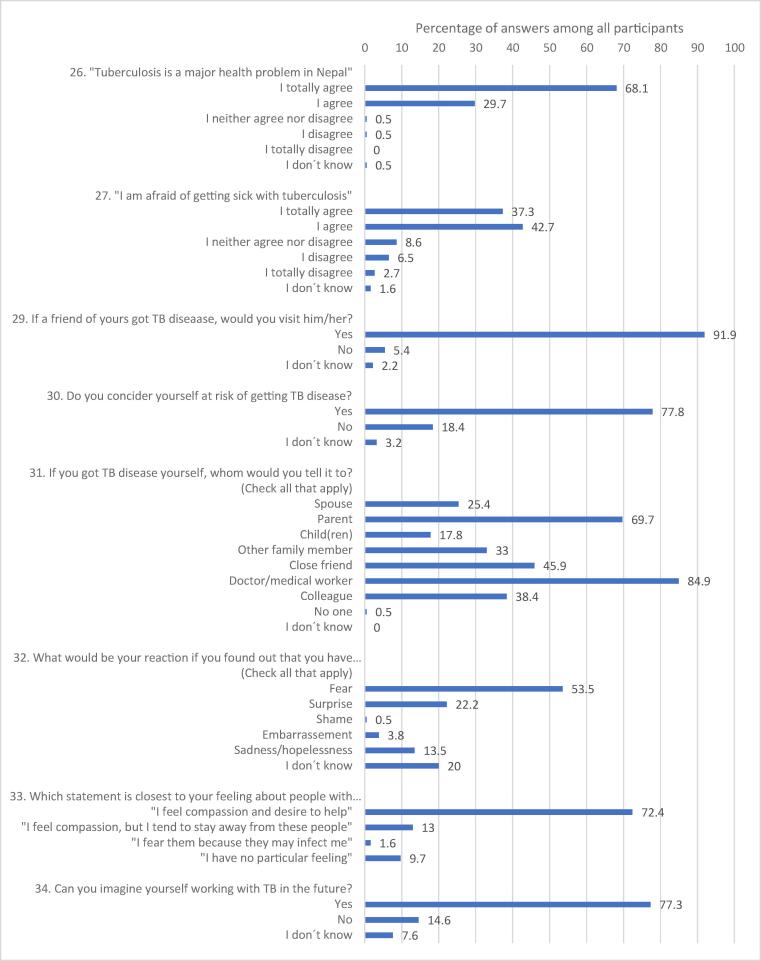
Distribution of answers on attitude questions.

Among the interns, 144 (77%) said they could imagine themselves working with TB patients in the future and 179 (97%) wanted to learn more about TB (Fig. 2). Altogether 144 (78%) interns considered themselves to be at risk of contracting the disease. If a friend of them developed TB, 171 (92%) of them would visit him/her. When asked about how they would react if they developed TB themselves, 99 (54%) of the interns said they would react with fear, 41 (22%) with surprise, 25 (14%) would feel sadness/hopelessness and 7 (4%) embarrassment (Fig. 2). More than 1/3 would only tell the diagnosis to a doctor/medical worker, and to nobody else.

The mean attitude score was 9.4 (SD:1.89) and CMS and NMC had a higher score than KUSMS (Table 2, Table 4). Being 25 years or older were found to have a positive association with the attitude score (Table 4). There was a positive correlation between greater knowledge and better attitude (0.3) p < 0,001 when tested using two tailed Pearson correlation test.

**Table 4:** Correlates of TB attitude, using one-way ANOVA.

	Variable	Estimate (95% CI)	P-value
Gender	Female vs. Male	0.2 (-0.4 to 0.7)	0.533
Age	≥25 vs. <25	0.7 (0.1 to 1.3)	0.015
Medical college	Overall		0.001
	CMS vs. KUSMS	1.2 (0.4 to 2.1)	0.002
	CMS vs. NMC	0.3 (-0.5 to 1.2)	1.000
	NMC vs. KUSMS	0.9 (0.1 to 1.6)	0.017
Tuberculin skin test	≤5 yrs vs. >5 yrs/never	0.3 (-0.4 to 1.0)	0.406
No. TB patients seen	≥10 vs. <10	0.3 (-0.4 to 0.9)	0.410
Friends or family with TB	No vs. Yes	0.1 (-0.5 to 0.7)	0.743
Knowledge score	Overall	0.3 (0.1 to 0.4)	<0.001

TST: Tuberculin skin test, CI: Confidence interval, KUSMS: Kathmandu University School of Medical Sciences, CMS: College of Medical Sciences, NMC: Nepal Medical College.

Test for normality and visual inspection of normal Q-q plots showed that the knowledge score was normally distributed. When adjusted for the knowledge score, the attitude score was also normally distributed in the same tests.

## 4 Discussion

### 4.1 Important findings

The WHO recommendations that medical school should “provide every graduate with the knowledge, skills and attitudes essential to the management of tuberculosis in the patient and in the community as a whole”[9] was only partly investigated in this study as the skills were not tested and not all details in the recommendations were checked. A large proportion of the interns were considered to have adequate knowledge overall regarding TB and the NTP, and there was no significant difference in the knowledge scores between the medical colleges included in the study. However, one should not ignore the identified knowledge gaps regarding at risk groups, symptoms and examination, and the current TB epidemiology in Nepal or the fact that 37% of the interns felt unable to speak freely of TB. There was a correlation between knowledge and attitude score. The median attitude score was considered adequate in general and 77% of the interns said they could imagine themselves working with TB in the future, while almost all of them acknowledged that TB was a major health problem in Nepal.

### 4.2 Knowledge

In contrast to some studies in other countries, which found insufficient TB related knowledge among medical students and/or interns [10], [11], [12], [13], [14], the present study found a proportion of approximately 70% correct answers witch was considered adequate TB knowledge among Nepalese interns in general. However, there are other studies that have found adequate knowledge [15], [16]. Like this study, those studies also found an association between better knowledge and the number of TB patients seen [15], [16]. All of the theoretical concepts had been taught according to the syllabus set by Kathmandu University, and there were no differences between the colleges in terms of the knowledge score. Students‘ knowledge could be improved by requiring them to spend some time at DOTS clinics and/or see a specified number of TB patients during medical school.

Even though the general knowledge was adequate, there were some serious knowledge gaps with resepct to at-risk groups, main symptoms of TB and sputum tests for both diagnosis and follow-up. Similar important knowledge gaps have been detected in other studies among both students/interns and medical doctors [11], [13], [19], [20]. These knowledge gaps could lead to serious mismanagement of TB patients, under- and over-diagnosis, and late diagnosis of MDR-TB. Twenty-seven percent of the interns did not know the standard treatment for TB, which is another risk for developing drug-resistant TB. Other studies of students and doctors have shown even higher numbers [10], [14], [16].

There was a positive association between having done a TST in the last five years and the knowledge score. In a study from Rome, where all students had done a TST, there was an association between the reported taking of the test and knowledge of TB. Other studies have also reported an association between knowledge and better-integrated preventive measures [21].

### 4.3 Attitude

Health care personnel’s attitude towards TB patients is perceived to be important for patients‘ treatment completion and health seeking behavior [13], [22]. In our study 77% of the interns could imagine themselves working with TB and 97% wanted to learn more about the disease. The mean attitude score was 9.4 of a maximum of 14 and was considered good. However, one-third of the interns felt unable to speak freely about TB. In a study of family physicians in Turkey, almost half of the doctors could not imagine themselves working with TB patients [20], while in a study of residents in India 51% reported fear, lack of compassion and a tendency to avoid TB patients [23]. Thus, the Nepalese interns in general, have better attitudes, which can serve as a good base for further improvements in knowledge and attitudes towards TB.

### 4.4 Strengths and limitations

As our questionnaire is based on the WHO‘s KAP template, which has been developed by skilled and knowledgeable developers [17], the questionnaire has an acceptable professional standing. In addition, the anonymity provided by the survey, which could prevent any negative individual consequences and could reduce the threshold for responding, resulted in a higher sample size. Most of the interns filled out the questionnaires completely, and just three responses had to be removed, while one was excluded from some of the analyses due to insufficient answers. These advantages provided a solid base for our analyses and survey.

The completion rate of the present study was good (69%) and no one declined to answer the questionnaire. However, we do not know exactly how many interns were asked to participate but were told that everybody who was asked, answered the questionnaire. The percentage of interns who participated varied from 49 to 90%.This could have been caused by handing out the questionnaires to only a fraction of the interns at the respective colleges. Due to the time limitation, the questionnaires were handed out to the interns present at the colleges at the time of the study which could have caused a selection bias. However, this bias would have been random and only diluted the results, not led the results in any particular direction. Furthermore, as it was a written questionnaire, some of the participants checked outside of the box, between two boxes, or in too many or too few boxes, all leading to an imprecise estimate of their KAP. As three medical colleges in different parts of Nepal were included, and their results are mostly in conformity, our results might extrapolate to the rest of the country.

Confounders, such as interest, clinical practice and personal experience, could have led to better participation in the survey yielding higher knowledge and attitude scores. The questionnaire was not filled out in a controlled environment, making it impossible to state if the interns used the internet, books or other sources for information. The consequence could be a falsely higher knowledge score. Generalisability could be suboptimal due to the low percentage of included interns, because all the medical colleges were affiliated to KU and none were government-run, and because only three out of 19 medical colleges were included in the study.

## 5 Conclusion

The surveyed interns had adequate knowledge level about TB, and acceptable attitudes towards the disease in general. However, some knowledge gaps and unacceptable attitudes were also found, including with respect to disease detection and management. As most of the interns could imagine themselves working with TB in the future, and are receptive to more education in this area, TB should be a priority area in medical education including in their post-graduate education. This survey underlines the importance of clinical experience and seeing TB patients during medical college. We suggest that all students gain experience at a TB clinic or see a minimum prescribed number of TB patients during medical schools.

## Ethical statement

The interns were given an information sheet and gave informed consent by participating in the study. The data was collected anonymously, and only unidentifiable sociodemographic data was collected.

A verbal request to Regional Committees for Medical and Health Research Ethics Norway clarified that ethical approval in Norway was not needed as no patient information was gathered in the study.

In Nepal ethical approval was received from KUSMS Institutional Review Committee (KUSMS/IRC) (84/17), at NMC the Research Director gave an approval from IRC-NMC, and CMS accepted the KUSMS/IRC.

## Declaration of Competing Interest

The authors declare that they have no known competing financial interests or personal relationships that could have appeared to influence the work reported in this paper.

## Appendix

Scoring system for calculating attitude score

Scoring system for calculating knowledge score

Questionnaire with information sheet and marked correct answers
